# How to Reduce the Incidence of Recurrence Following Catheter Ablation for Atrial Fibrillation

**DOI:** 10.1002/clc.70302

**Published:** 2026-04-11

**Authors:** Naoya Kataoka, Teruhiko Imamura

**Affiliations:** ^1^ Second Department of Internal Medicine University of Toyama Toyama Japan

## Conflicts of Interest

The authors declare no conflicts of interest.

To the Editor,

The recurrence of atrial fibrillation (AF) following catheter ablation remains an unresolved clinical issue. The authors developed a novel risk model to predict AF recurrence by integrating electro‐anatomical voltage mapping with hematological and inflammatory biomarkers [1]. This approach is innovative and clinically relevant; however, several points warrant further discussion.

Although multiple biomarkers have been proposed to predict AF recurrence—including inflammatory markers, as highlighted in the present study [1], as well as oxidative stress–related biomarkers [2]—the precise causal relationship between these biomarkers and AF recurrence remains unclear. In particular, whether these circulating biomarkers directly reflect atrial substrate remodeling or merely represent epiphenomena associated with advanced disease is uncertain. An analysis exploring the correlation between biomarker levels and the extent of low‐voltage areas might further clarify the mechanistic link between systemic inflammation and atrial structural remodeling.

The ultimate goal of catheter ablation for AF extends beyond rhythm control to the prevention of clinically meaningful outcomes, such as heart failure exacerbation and thromboembolic events. In this context, baseline data on thrombotic and bleeding risks—assessed using established scores such as CHA₂DS₂‐VASc and HAS‐BLED—would be informative. Evaluating whether the proposed novel risk score is associated not only with AF recurrence but also with these downstream clinical outcomes would further strengthen its clinical utility.

An important unanswered question is how AF recurrence can be reduced in patients identified as high risk by this model. Our group has previously demonstrated that impaired mitochondrial function, particularly defective mitochondrial autophagy, is associated with AF recurrence after catheter ablation [3]. From this perspective, therapeutic strategies targeting mitochondrial dysfunction may represent a promising adjunctive approach. For example, metformin has been reported to improve mitochondrial function and may reduce AF recurrence even in patients without diabetes mellitus. Incorporating such mechanistically driven interventions into future studies could provide a more comprehensive strategy for personalized AF management.

## Data Availability

The authors have nothing to report.
